# Fibrin clot and Leukocyte-rich platelet-rich fibrin show similar release kinetics and amount of growth factors: a pilot study

**DOI:** 10.1186/s13018-023-03709-5

**Published:** 2023-03-25

**Authors:** Yuta Nakanishi, Takehiko Matsushita, Kanto Nagai, Daisuke Araki, Yuichi Hoshino, Ryosuke Kuroda

**Affiliations:** Department of Orthopaedic Surgery, Kobe University Graduate School of Medicine, 7-5-1 Kusunoki-Cho, Chuo-Ku, Kobe, 650-0017 Japan

**Keywords:** Fibrin adhesive, Platelet-rich fibrin, Meniscus, Growth factor, Fibrin clot

## Abstract

**Background:**

In knee arthroscopic surgery, fibrin clot (FC) and leukocyte-rich platelet-rich fibrin (L-PRF) may be used in augmentation for meniscal repair. Studies have investigated growth factors released from FC and L-PRF; however, it is difficult to compare FC and L-PRF between different studies. Direct comparison of growth factors that may support meniscal healing released from FC and L-PRF may be beneficial in deciding whether to use FC or L-PRF. If no significant difference is seen, the surgeon may decide to use FC which is easier to prepare compared to L-PRF. The purpose of this pilot study is to investigate the release amount and pattern of basic fibroblast growth factor (bFGF), platelet-derived growth factor AB (PDGF-AB), transforming growth factor β1 (TGF-β1), vascular endothelial growth factor (VEGF), and stromal cell-derived factor 1 (SDF-1) from FC and L-PRF.

**Method:**

Twenty milliliters (ml) of whole blood was collected from each of the four volunteers. Ten milliliters of whole blood was allocated for preparation of FC and 10 ml for L-PRF. FC and L-PRF were separately placed in 5 ml of culture media. Five milliliters of the culture media was sampled and refilled at 15 min, 1 day, 3 days, 1 week and 2 weeks. The collected culture was used to quantify bFGF, PDGF-AB, TGF-β1, VEGF, and SDF-1 release by Enzyme-linked immune-sorbent assay (ELISA). Mann–Whitney U test was performed to assess significance of differences in amount of each growth factor released between FC and L-PRF. Significance was accepted at *P* value less than 0.05.

**Results:**

At two weeks, the cumulative release of TGF-β1 was the highest among all the growth factors in both FC and L-PRF (FC:19,738.21 pg/ml, L-PRF: 16,229.79 pg/ml). PDGF-AB (FC: 2328 pg/ml, L-PRF 1513.57 pg/ml) had the second largest amount, followed by VEGF (FC: 702.06 pg/ml, L-PRF 595.99 pg/ml) and bFGF (FC: 23.48 pg/ml, L-PRF 18.2 pg/ml), which order was also common in both FC and L-PRF. No significant difference in final release amount and pattern was seen between FC and L-PRF.

**Conclusion:**

The current pilot study showed that cumulative release amount and release pattern of PDGF-AB, VEGF, TGF-β1, and bFGF did not significantly differ between FC and L-PRF during the two weeks of observation.

## Introduction

**Fig. 1:**
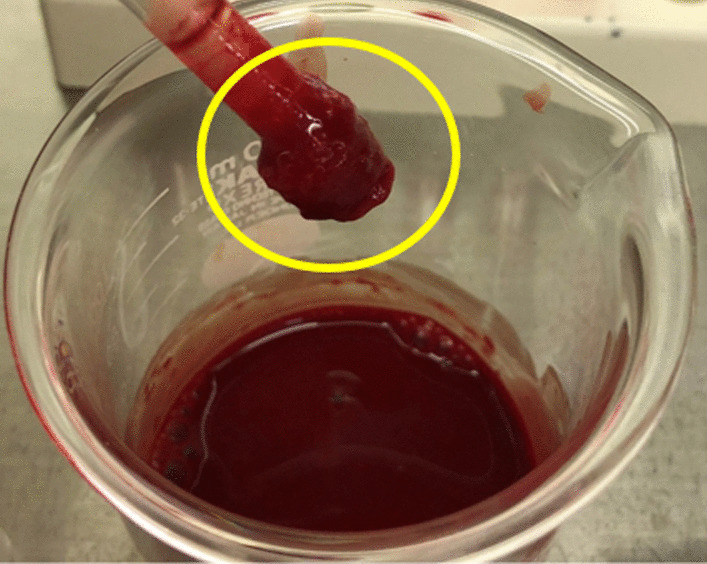
Fibrin clot formed around glass rod after stirring in whole blood. Yellow circle indicated the fibrin clot

**Fig. 2:**
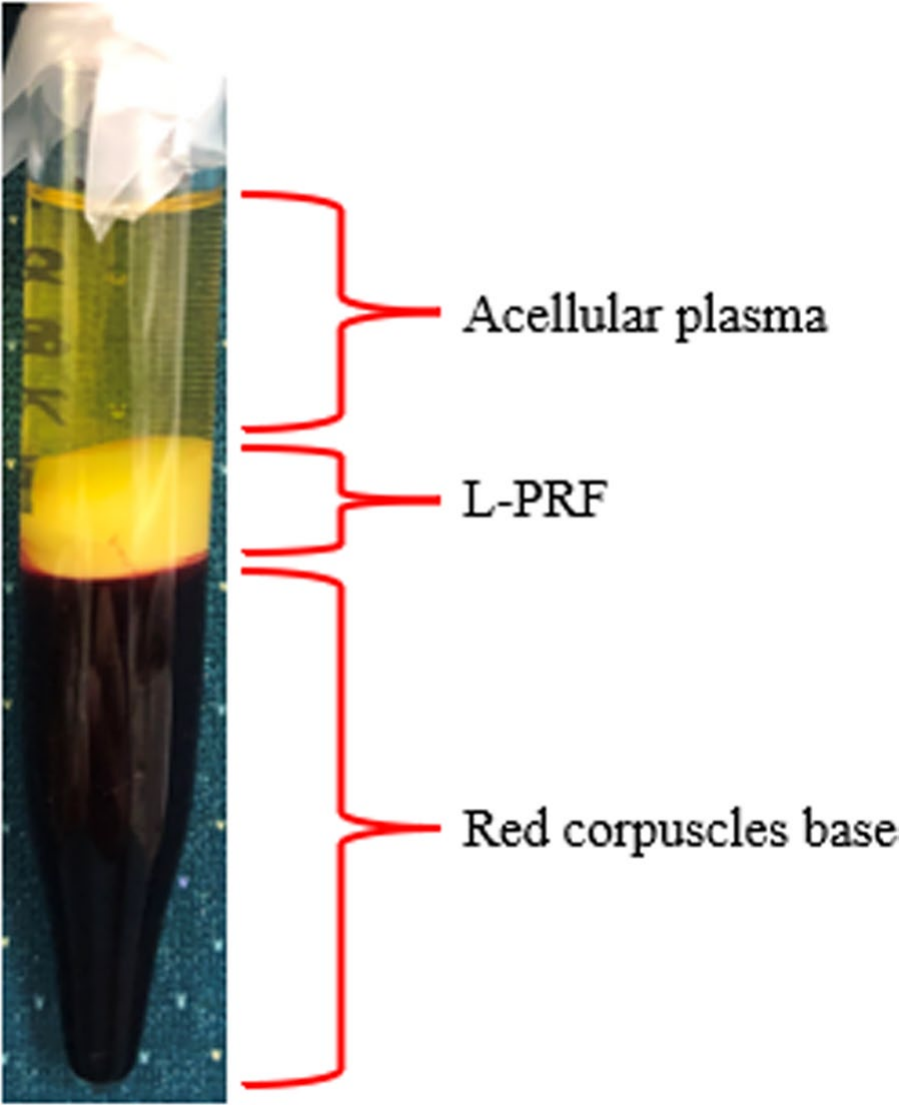
Three layers formed after centrifugation of whole blood. L-PRP is formed in the middle layer

The meniscus primarily functions to efficiently transmit load through the femoro-tibial joint and provides chondral protection for the femoral and tibial joint surfaces [1]. Therefore, injury or resection of the meniscus causes increase in contact area and pressure of femoral and tibial chondral surfaces [2]. This has been shown to cause progression of osteoarthritis [3]. Therefore, in the current clinical practice, meniscal repair is preferred over resection for indicated cases. A successful repair relies on intervention providing a favorable biomechanical and biological environment for healing. Significant improvement in biomechanical stability has been achieved by recent development of novel suture configurations [4]. However, success and widespread use of biologics in meniscal repair is still limited (Figs. 1, 2).

Autologous fibrin clot (FC) made from peripheral blood has been reported to contain growth factors and a fibrin complex advantageous in tissue regeneration in the meniscus [5]. Promising clinical outcomes have been reported with use of fibrin clots in combination with meniscal repair [6]. Recently, platelet concentrates such as platelet-rich plasma (PRP) and platelet-rich fibrin (PRF) have regained attention. Platelet concentrates can be divided into four groups; pure PRP (P-PRP), leucocyte-rich PRP (L-PRP), pure PRF (P-PRF), and leucocyte-rich PRF (L-PRF) [7]. PRP may be an effective biological augmentation to improve outcome when combined with meniscal repair [8]. However, concern lies in the use of anticoagulants, addition of activation agents, and bovine thrombin products which may have inherent risk of allergic reactions or infection, and widespread use may be hindered due to high cost [7]. P-PRF also utilized a kit that needs to be purchased at substantial cost and requires an anticoagulant and a specific separatory gel to remove leukocytes and attain high concentrate of platelets [7]. Conversely, FC and L-PRF are attractive in that they can be produced in the absence of anticoagulants or any foreign or artificial agents at cheap costs, allowing for widespread use without the concern of cost and regulations regarding injection or application of additive agents to patients [9]. FC can be made by simply stirring blood taken from the patient [10]. L-PRF similarly utilizes blood from patients but requires centrifugation [11]. FC and L-PRF have been shown to release growth factors such as basic fibroblast growth factor (bFGF), platelet-derived growth factor AB (PDGF-AB), transforming growth factor β1 (TGF-β1), vascular endothelial growth factor (VEGF), and stromal cell-derived factor 1 (SDF-1) [12–14]. Each growth factor has been shown in previous basic research to possess the potential for chemotactic and mitogenic properties favorable in meniscal healing [15–20]. However, great discrepancy still exists for data on L-PRF and scarcity in studies for FC that analyze release kinetics and release amount of growth factors. Therefore, there is lack of evidence for surgeons to decide whether FC or L-PRF should be used. If no significant difference is seen, the surgeon may decide that an easily prepared FC may suffice.

The aim of this pilot study is to elucidate release pattern of growth factors, PDGF-AB, VEGF, TGF-β1, bFGF, and SDF1 and compare between FC and L-PRF.

## Methods and materials

### Blood sample

Four samples of blood were collected from four volunteers. Informed consent was obtained from each volunteer and approved by the ethics committee (The Institutional Review Board of Kobe University ID No. B200232). Volunteers included were to have no history of medication or disease that may interfere with coagulation, no history of smoking, and normal concentration of white blood cells, red blood cells, and platelets. Due to the small number of subjects because of limitation in enzyme-linked immune-sorbent assay (ELISA) test kits available, male subjects in their 30 s were chosen, so the data collected from the subject can be analyzed with mitigated effect of age and sex. Also, this subject age was chosen as they would be the typical patient age group that would be candidates for meniscal repair with biological augmentation. All blood samples were collected in the morning before meal since white blood cells and platelets, that affect growth factor release, have been reported to be affected by food intake [21, 22]. Twenty milliliters of whole blood was taken from each volunteer. Ten milliliters was allocated to prepare FC and the remaining ten milliliters to prepare standard L-PRF.

### Fibrin clot (FC)

FC was prepared by gently hand stirring ten milliliters of blood in a sterile 250 ml beaker with a glass stir rod for approximately ten minutes [10]. After a clot is formed around the stir rod, the clot is removed from the beaker and placed in a 6-well in vitro plastic culture dishes with 5 cc of culture media (Dulbecco’s modified eagle’s medium with L-glutamine) without addition of fetal bovine serum [14].

### Leukocyte-rich platelet-rich fibrin (L-PRF)

L-PRF was isolated as previously described [23]. Briefly, ten milliliters of whole blood without anticoagulant was put into a glass tube, centrifuged at 2700 rotation per minute (rpm) (325 g) for 12 min. After centrifugation, three layers are formed with the middle layer containing L-PRF, between the red corpuscles at the bottom and acellular plasma at the top. L-PRF clot was removed with sterilized tweezers and separated from the red blood cell base using sterilized scissors [24]. Similar to FC, the L-PRF clot was placed in 6-well in vitro plastic culture dishes with 5 cc culture media.

### Measurement of growth factors

The dishes were incubated at 37 ℃, 5% carbon dioxide (CO2) [21]. The amount of growth factors released in the culture media were sampled at 15 min, 1 day, 3 days, 7 days and 14 days [14, 21]. In reference to a previous study, 5 ml of culture media was collected at each time point, frozen at − 80 ℃ and replaced with 5 ml of additional culture media [14]. Measurement of growth factors was performed by ELISA kits (Luminex® Assay Human Premixed Multi-Analyte Kit, R&D Systems, Inc., Minneapolis, USA and TGF-β1 Single Plex Magnetic Bead Kit, EMD Millipore, Darmstadt, Germany). ELISA was performed at Filgen, Inc. (Nagoya, Japan). PDGF-AB, VEGF, TGF-β1, bFGF, and SDF1 were chosen to be analyzed since the growth factors have been shown to be favorable in meniscal healing and have been detected to be released from platelet concentrates [12, 13].

### Statistical analysis

Mann–Whitney U test was performed to assess significance of differences in amount of each growth factor released between FC and L-PRF. Significance was accepted at P value less than 0.05 (GraphPad Prism version 9.0.2 for Windows, GraphPad Software, San Diego, CA, USA).

## Results

Patient age, sex, red blood cell, white blood cell, and platelet counts between each patient are reported in Table 1. All subjects had normal blood count.

**Table 1:** Age, sex, red blood cell (RBC), white blood cell (WBC), and platelet (PLT) count of included volunteers

	Sub. 1	Sub. 2	Sub. 3	Sub. 4
Age (y.o.)	36	34	35	35
Sex	M	M	M	M
BMI	25.9	24.5	25.5	23.7
RBC (g/dl)	505	523	515	528
WBC (/μl)	3790	5260	5200	4100
PLT (10,000/μl)	26.9	21.3	22.2	23.7

### Growth factor release at each time point

**Fig. 3:**
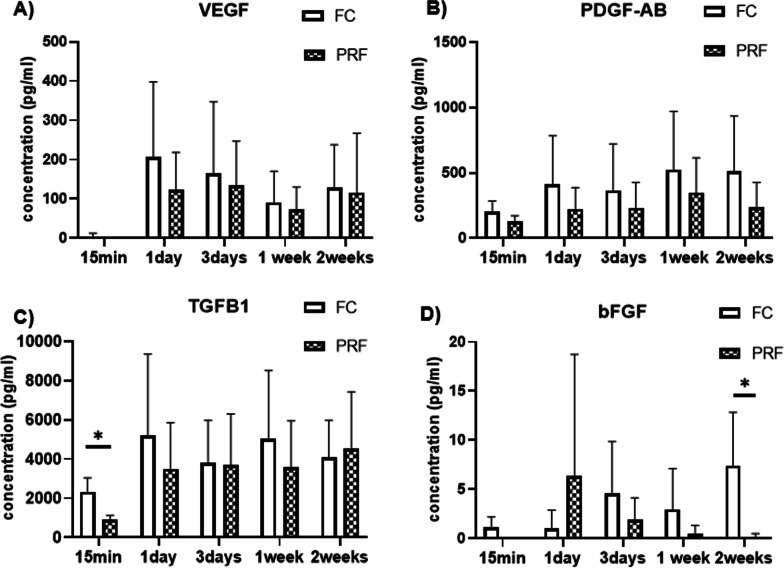
Growth factor release at each time point quantified by ELISA. **A**–**D** shows growth factor release at each time point for PDGF-AB, VEGF, TGFβ1, and FGF, respectively. Error bar indicates standard deviation. * indicates statistical significant set at *P* < 0.05

The amount of growth factor released at each time point for FC and L-PRF is shown in Fig. 3. The amount of TGF-β1 released at 15 min was significantly higher for FC. FC showed the greatest percentage of TGF-β1 release at day 1, whereas L-PRF showed the greatest percentage of release at day 3. Both FC and L-PRF showed a second peak, at 1 week for FC and at 2 weeks for L-PRF. FC had significantly higher amount of bFGF release at 2 weeks. The largest percentage of bFGF release occurred at day 1 for PRF (71.1%), while the highest percentage of bFGF release was greatest at 2 weeks for FC (43.4%). PDGF-AB, VEGF showed no significant difference in amount released at each time point between FC and L-PRF. The amount of VEGF released at each time point peaked at day 1 for FC and at 3 days for PRF. Both FC and L-PRF showed increase between 1 and 2 weeks. ELISA could not detect the release of SDF1 in most of the samples, rendering it impossible to attain any results for amount released at each time point.

### Cumulative release of growth factors

**Fig. 4:**
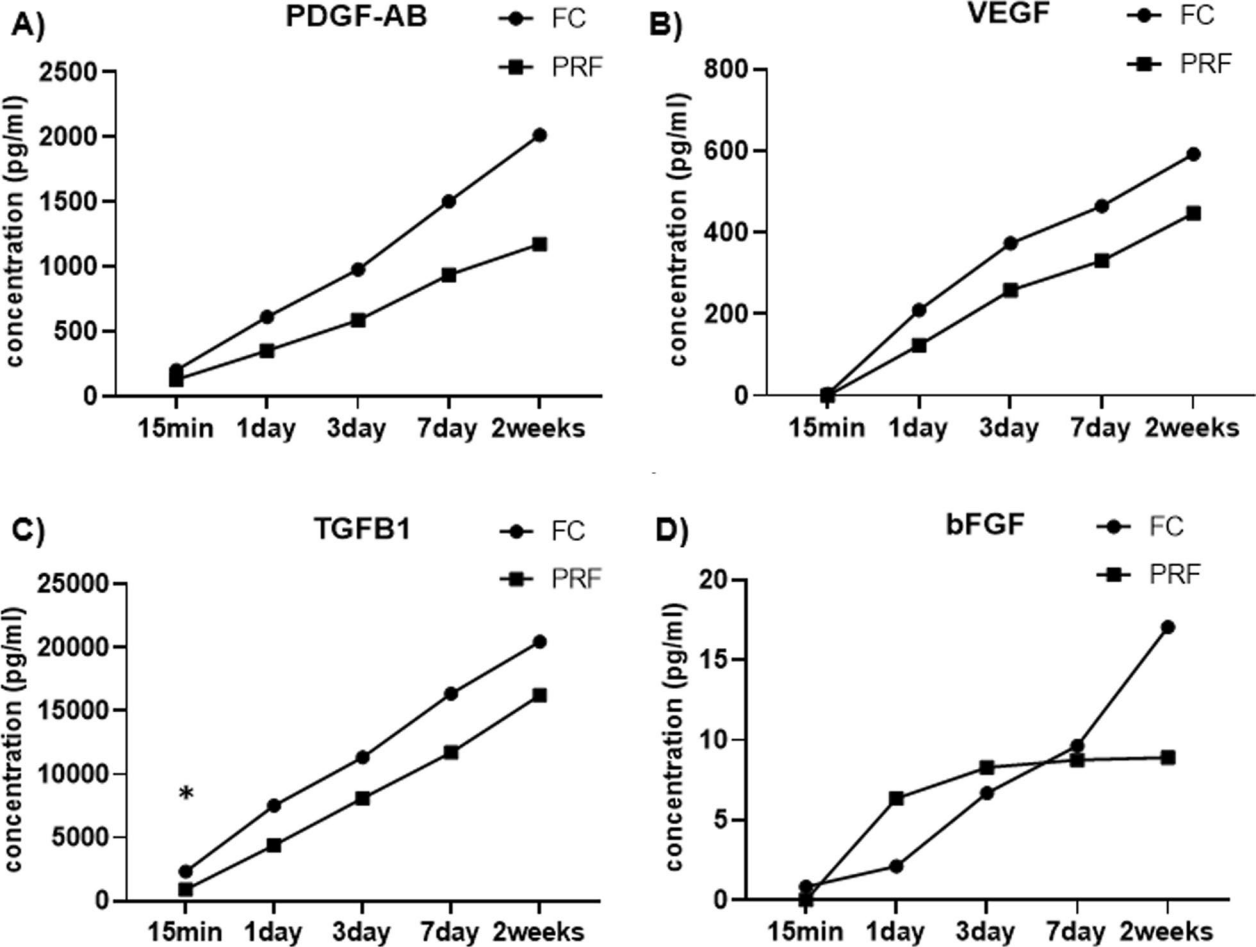
Cumulative growth factor release at each time point quantified by ELISA. **A**–**D** shows growth factor release at each time point for PDGF-AB, VEGF, TGFβ1, and FGF, respectively. *Indicates statistical significant set at *P* < 0.05

The cumulative amount of growth factors released after 2 weeks is shown in Fig. 4. At two weeks, the release of TGF-β1 was the highest among all the growth factors (FC:19,738.21 pg/ml, L-PRF: 16,229.79 pg/ml) in both FC and L-PRF. PDGF-AB (FC: 2328 pg/ml, L-PRF 1513.57 pg/ml) had the second largest amount, followed by VEGF (FC: 702.06 pg/ml, L-PRF 595.99 pg/ml) and bFGF (FC: 23.48 pg/ml, L-PRF 18.2 pg/ml), which order was also common in both FC and L-PRF.

The cumulative release amount of TGF-β1 at 15 min was significantly higher in the FC group. However, no statistically significant difference between FC and L-PRF was seen for cumulative amount of PDGF-AB, VEGF, TGF-β1, and bFGF released at two weeks. SDF1 was not detected in most of the samples, rendering it impossible to attain any results for cumulative amount released.

## Discussion

The most important finding in this study was that the cumulative release amount and release kinetics of PDGF-AB, VEGF, TGF-β1, and bFGF did not significantly differ between FC and L-PRF during the two weeks of observation. Additionally, continuous release of PDGF-AB, VEGF, TGF-β1 was seen even at the 2 weeks observation point for both FC and L-PRF.

When comparing FC and L-PRF, although not statistically significant, the current study showed higher cumulative amount of TGF-β1, bFGF, VEGF, and PDGF-AB in FC. Schar et al. also found higher amount of cumulative TGF-β1 and VEGF in natural blood clot compared to L-PRF [21]. Similarly, Varelo et al. found VEGF to be higher in blood clot compared to L-PRF [25]. In agreement with Schar et al., this is suggestive of possible loss of leukocyte and platelet during production of L-PRF, which are known to release growth factors [21]. Growth factor concentration is known to be higher at the red-yellow surface (bottom of part) of L-PRF caused by the gravity from centrifugation [13]. Therefore, centrifugation may have caused some leukocytes to be forced further to the bottom of the tube away from the L-PRF causing decreased number of leukocytes within L-PRF.

The release kinetics show continued release of the growth factors for 2 weeks in both FC and L-PRF with a similar pattern. Previous studies have also shown blood clot and L-PRF to have growth factors release continuing up to 28 days [14, 21]. It is interesting to see TGF-β1 and VEGF shared a pattern of release in which VEGF at day 7 and TGF-β1 at day 3–7 show a trough in amount of release, but after, show increase in amount released similar to the study by Schar et al. [21] This is in line with previous studies that the sustained release of TGF-β1 and VEGF may be due to production by leukocytes captured in the fibrin matrix [21, 26]. When the level of those two growth factors becomes too low, leucocytes could produce new molecules to support a necessary level of these growth factors [27].

In the clinical setting, considering that approximately 10% of total amount released for PDGF-AB and TGF-β1 are released within the first 15 min after procurement, application of FC and L-PRF should be performed immediately to maximize the use of the growth factors. In addition, L-PRF retained its structure compared to FC after the 2-week study period. This structural integrity of L-PRF may be advantageous to serve as a scaffold in meniscal healing better than FC.

Other than the use of FC or L-PRF prepared from peripheral blood, microfracture of the intercondylar notch has also been reported to enhance meniscal healing by growth factor release intra-articularly via bleeding from the bone marrow [28]. Hashimoto et al. compared release amount of bFGF, TGF-β, VEGF, PDGF-AA, and SDF-1, between bone marrow aspirate (BMA), and peripheral blood in both clotted and unclotted forms. The study showed bFGF, TGF-β, and SDF-1 levels in BMA clots were higher compared to peripheral blood clots. Conversely, as for PDGF-AA, the concentration was lower in BMA clots compared to peripheral blood clots. Also, except for PDGF-AA, no significant differences were seen between BMA and peripheral blood in the unclotted form. Therefore, in respect to growth factor release, a definite difference between microfracture and use of FC and L-PRF made from peripheral blood cannot be made.

It is difficult to directly compare release amount of growth factors from FC and L-PRF with previous studies since there is no consensus on protocol for production of FC or L-PRF, and experimental designs vary between studies. Centrifugation speed can influence organization and the size of fibrin fibers, and also shape and viability of cells in the fibrin matrix [29]. Blood collection volume, human variability, and differences in preparation method also affect the amount of growth factor released [30]. Even intra-subject variation in cell type and count with repetitive blood draws are shown to cause difference in amount of growth factor release [30]. As such, the many variables which may contribute to the amount of growth factor released may explain the high standard deviation in the amount of growth factors released by FC and L-PRF. Moreover, the amount of factor release is in the scale of picogram or nanogram level, subjecting minor discrepancies in procedure and experimental design to effect measured amounts. However, although comparison of exact values from the current study to previous studies may not be of great importance, measured amount of growth factor release in the current study is reflective of previous studies with similar protocol which can support the legitimacy of experimental technique in this study [5, 14, 21]. It is difficult to give a definitive reason as to why secretion level of SDF-1 was so small in this study even though it has been shown that SDF-1 can be found in fibrin clot made from peripheral blood [31]. One reason may be that, although fibroblasts are shown to release SDF-1 [32], the main source is in the bone marrow [31], which could explain why almost no release was seen in the current study which only utilized peripheral blood.

Limitation of this study is that it is an in vivo study in which FC and L-PRF samples were cultured in an artificial environment. An in vivo environment such as the knee upon meniscal repair will expose FC and L-PRF to joint fluid and bleeding from surgical procedure and mechanical stress which may influence growth factor release and patterns. However, the advantage of an in vitro study is that conditions can be better controlled to compare FC and L-PRF in a similar environment. As a result, it showed that significant difference in release amount or pattern of growth factors may not exist between FC and L-PRF. The sample size of this study was limited due to the cost of the ELISA kits. This may have caused type II error in statistical analysis. Due to the limitation in sample size, subjects were chosen to be men in their thirties to reflect the common patient population that receive meniscal repair with biological augmentation. Also, by choosing patients in the same age group mitigates the factor of age effecting the results in this study since previous study has suggested that difference in age may cause difference in minimum and maximum growth factor accumulation [14]. However, if greater number of samples can be tested, it will be ideal to have subjects from different age groups and different sex to compare release amount and pattern to decipher any differences that can aid in clinical decision making.

## Conclusion

The current pilot study showed that cumulative release amount and release pattern of PDGF-AB, VEGF, TGF-β1, and bFGF did not significantly differ between FC and L-PRF during the two weeks of observation. The current results suggest that, in respect to ease of production and low cost, FC may be preferred over L-PRF as biological augmentation. However, L-PRF may be preferred if a scaffold that can maintain structural integrity for a longer duration is required. In vivo studies with a larger sample size are needed to elucidate clinical efficacy.

## Data Availability

The data presented in this study are available from the corresponding author on reasonable request.

## Abbreviations

bFGF: Basic fibroblast growth factor
BMA: Bone marrow aspirate
ELISA: Enzyme-linked immune-sorbent assay
FC: Fibrin clot
L-PRF: Leukocyte-rich platelet-rich fibrin
L-PRP: Leucocyte-rich platelet-rich plasma
PDGF-AB: Platelet-derived growth factor AB
PRF: Platelet-rich fibrin
PRP: Platelet-rich plasma
SDF-1: Stromal cell-derived factor 1
TGF-β1: Transforming growth factor β1
VEGF: Vascular endothelial growth factor
